# A Comprehensive Review of Bioactive Constituents, Health-Promoting Effects, Processing Technologies, and Industrial Applications of *Sparassis crispa* Sensu Lato

**DOI:** 10.3390/foods15122152

**Published:** 2026-06-14

**Authors:** Xin Chen, Yunzhe Guo, Xiaotong Dong, Yujin Cao, Min Xie, Yibin Li, Li Wu

**Affiliations:** 1 College of Food Science, Fujian Agriculture and Forestry University, Fuzhou 350002, China; cx15160514825@163.com (X.C.); 17831004058@163.com (Y.G.); 15847490420@163.com (X.D.); 15059806533@163.com (Y.C.); 18960978734@163.com (M.X.); 2 Institute of Food Science and Technology, Fujian Academy of Agricultural Sciences, Fuzhou 350003, China; 3 National R&D Center for Edible Fungi Processing, Fuzhou 350003, China; 4 Key Laboratory of Subtropical Characteristic Fruits, Vegetables and Edible Fungi Processing (Co-Construction by Ministry and Province), Ministry of Agriculture and Rural Affairs, Fuzhou 350003, China; 5 Fujian Province Key Laboratory of Agricultural Products (Food) Processing Technology, Fuzhou 350003, China

**Keywords:** *Sparassis crispa*, bioactive constituents, health benefits, processing technologies, industry development

## Abstract

*Sparassis crispa* sensu lato (*S. crispa*) is a highly valued medicinal and culinary fungus rich in nutritional and bioactive compounds. Polysaccharides, particularly β-glucans, are its most extensively studied constituents, accounting for up to 43.6% of its dry weight—the highest concentration reported among edible fungi. Alongside proteins, terpenoids, phenolics, and ergosterol, these biomolecules confer diverse physiological benefits, including immunomodulatory, antitumor, antioxidant, anti-inflammatory, and neuroprotective effects, as well as the capacity to modulate gut microbiota. Consequently, *S. crispa* exhibits substantial market potential and is increasingly incorporated into functional foods, nutraceuticals, pharmaceuticals, and cosmetics. This review systematically summarizes the primary bioactive components of *S. crispa* sensu lato and their associated health benefits, with emphasis on recent advances in immunomodulatory, antitumor, and antioxidant activities. Furthermore, it critically compares the retention of active ingredients, product quality, and bioavailability of key processing technologies. These technologies include various drying methods, grinding techniques, extraction methods, and formulation systems. Despite significant research progress, challenges persist regarding optimal cultivation conditions and standardized industrial processing. Future perspectives highlight the necessity for intelligent cultivation strategies, the adoption of advanced processing technologies, and robust policy support to drive the sustainable development and commercial exploitation of the *S. crispa* industry.

## 1. Introduction

*Sparassis crispa* sensu lato *(S. crispa),* commonly known as the “cauliflower mushroom” due to its distinctive frond-like fruiting body, is an emerging medicinal and culinary fungus belonging to *Basidiomycota*, *Agaricomycetes*, *Polyporales*, *Sparassidaceae*, and *Sparassis* [1]. It is widely distributed across northern temperate regions—spanning Eastern North America, Europe, and Asia [2]. *S. crispa* comprises multiple distinct species, including *Sparassis latifolia* (*S. latifolia*) and *Sparassis subalpina (S. subalpina)*. When citing specific studies, the original species name reported in the literature (*S. latifolia* or *S. subalpina*) is retained. These taxa share a close phylogenetic relationship, high morphological similarity, and largely overlapping chemical profiles. Therefore, data derived from *S. latifolia* and *S. subalpina* are considered highly relevant to *S. crispa* and generally transferable for this review [3].

Culturally revered in Japan as the “dream mushroom” for its desirable texture and profound health-promoting properties [4], *S. crispa* is recognized as a rich source of essential macronutrients and micronutrients. Notably, it boasts an exceptional concentration of β-glucans (up to 43.6% of its dry weight), representing the highest level reported among edible fungi [5,6]. Consequently, extensive research has demonstrated its broad spectrum of physiological benefits, encompassing immunomodulatory, antitumor, antioxidant, neuroprotective, anti-inflammatory, and antimicrobial activities, alongside wound-healing, anti-obesity, and antihypertensive effects [7,8,9,10,11,12,13]. To effectively harness these valuable bioactive components for consumer products, appropriate post-harvest processing is critical. Traditional processing methods, such as high-temperature drying, hot-water extraction, and coarse pulverization, have primarily focused on extending shelf life and improving flavor. However, these methods often cause thermal degradation of heat-sensitive bioactive compounds and exhibit relatively low extraction efficiency. In recent years, advanced processing technologies—including vacuum freeze-drying, ultrafine pulverization, ultrasound-assisted extraction, and ultra-high-pressure extraction—have been increasingly adopted to better preserve heat-labile components and enhance the retention and bioavailability of key bioactives such as polysaccharides and phenolics.

Currently, there is a lack of comprehensive literature that systematically bridges its bioactive profiling with modern processing technologies and industrial applications. Therefore, this review aims to systematically summarize the nutritional composition and diverse health benefits of *S. crispa* sensu lato, critically compare the effects of different drying methods and extraction techniques on the retention of bioactive compounds and overall product quality, and outline current industrial challenges. Ultimately, this review seeks to provide a robust scientific foundation and future perspectives for the sustainable development and high-value utilization of *S. crispa* in the functional food and nutraceutical industries.

## 2. Literature Search Strategy

Relevant literature was systematically retrieved from multiple databases, including Web of Science, PubMed, Scopus, China National Knowledge Infrastructure, and Google Scholar, up to December 2025. Search strategies combined the following keywords and their synonyms using Boolean operators (AND/OR): “*Sparassis crispa*”, “*Sparassis latifolia*”, “*Sparassis subalpina*”, “*Cauliflower mushroom*”, “*Hanabiratake*”, together with terms related to bioactive components (“β-glucan”, “polysaccharide”, “bioactive compounds”, “phenolics”, and “ergosterol”), health effects (“immunomodulatory”, “antitumor”, “antioxidant”, “anti-inflammatory”, “neuroprotective”, “health benefits”), and processing technologies (“drying”, “extraction”, “ultrasound-assisted”, “ultra-high pressure”, “processing”, and “deep processing”). Studies were selected if they directly reported the chemical composition, structural characterization, biological activities, processing methods, or industrial applications of *S. crispa*. When evidence from distinct species (*S. latifolia* or *S. subalpina*) was included, the original species name was explicitly retained in the text, and data were discussed in the context of the *S. crispa* sensu lato complex due to their high morphological, phylogenetic, and chemical similarities. Both original research articles and review articles were included. Duplicate publications, conference abstracts without full text, and studies lacking sufficient methodological details were excluded.

## 3. Nutritional and Bioactive Composition

*S. crispa* is a nutritionally dense medicinal and culinary fungus characterized by a low fat content, a high proportion of unsaturated fatty acids, and an abundance of functional constituents, including polysaccharides, proteins, sterols, phenolics, and terpenoids. The synergistic presence of these bioactive substances underpins their unique medicinal properties [14,15], demonstrating significant potential for health promotion and disease prevention.

### 3.1. Polysaccharides

Polysaccharides from *S. crispa* have garnered extensive attention due to their notable biological activities, particularly their immunomodulatory, antitumor, and antioxidant effects. These complex macromolecules are primarily composed of monosaccharides such as glucose, galactose, fucose, and mannose [2]. As the principal active constituents, both their structural characteristics and biological functions remain at the forefront of current research. Notably, β-glucan constitutes up to 43.6% of the dry weight of *S. crispa* [16]. This specific glucan predominantly exists as a 6-branched β-(1→3)-D-glucan. Structurally, its backbone consists of glucose units linked via β-(1→3)-glycosidic bonds, with branching occurring approximately every third glucose residue via β-(1→6)-glycosidic linkages [17]. This unique structural conformation is highly correlated with its broad spectrum of biological activities, including immunomodulatory, antitumor, antioxidant, and antimicrobial effects.

In recent years, significant progress has been made in characterizing functional polysaccharides from *Sparassis* species, providing a theoretical foundation for developing novel functional foods and therapeutics. The main characteristics of these polysaccharides are summarized in Table 1.

### 3.2. Proteins and Peptides

Beyond polysaccharides, *S. crispa* is a valuable source of functional proteins and peptides. It is rich in essential amino acids, including lysine, leucine, isoleucine, and phenylalanine, which are crucial for maintaining metabolic homeostasis, facilitating tissue repair, and supporting immune function. Recent studies have demonstrated that total protein extract (SLTP) from *S. crispa* exhibits significant inhibitory effects against gastrointestinal cancer cells, highlighting its antitumor potential [25]. Furthermore, a specific glycoprotein isolated via ultrasound-assisted salt extraction has been confirmed to possess substantial antioxidant activity [26]. Additionally, the successful isolation of a novel lectin from *S. crispa* has revealed potent antimicrobial properties [27].

### 3.3. Sterols, Terpenoids, and Phenolics

#### 3.3.1. Sterols

Sterols, predominantly ergosterol, represent a critical class of lipophilic bioactives in *S. crispa*. As a fundamental component of the fungal cell membrane, ergosterol exhibits antioxidant, anti-inflammatory, and antitumor properties. Importantly, as a provitamin, it can be converted into vitamin D_2_ upon ultraviolet (UV) irradiation, significantly enhancing the nutritional profile of the fungus [28,29]. Researchers have successfully isolated two ergosterol peroxides from *S. crispa*, identified as (3β,5α,8α,22E)-5,8-epidioxyergosta-6,22-dien-3-ol and 3-O-β-D-glucopyranosyl ergosterol peroxide [30]. Moreover, sequential chromatography facilitated the isolation of an ergosterol derivative, structurally determined as 5α,6α-epoxy-(22E,24R)-ergosta-8(14),22-diene-3β,7β-diol [31].

#### 3.3.2. Phenolics

Phenolic compounds contribute substantially to the antioxidant capacity of *S. crispa*. Studies have demonstrated that ultrasound-assisted extraction (UAE) utilizing a 50% aqueous methanol solvent effectively maximizes the total phenolic content (TPC) from *S. latifolia*. Comprehensive analysis of the methanolic extract of *S. crispa* (CES) identified five key phenolic compounds, all of which exhibited pronounced antioxidant activities, suggesting their primary role in neutralizing free radicals [10]. Additionally, a specific phenolic acid, 5-hydroxy-7-methoxyphthalide, was successfully isolated [32]. Furthermore, a 70% ethanolic extract of *S. crispa*, rich in highly hydrophobic polyphenols, has demonstrated promising potential in improving hair quality and health [33].

#### 3.3.3. Terpenoids

Terpenoids constitute one of the largest and most structurally diverse groups of natural products, widely distributed across flora and mycobiota [34]. Recent investigations have successfully identified various sesquiterpenoids and other distinct terpenoid derivatives (e.g., ainsliatone A and clitocybulol C) from this fungus [35]. Although several terpenoids have been isolated, current research remains predominantly focused on their preliminary identification and quantitative analysis. Given that the literature extensively documents the antioxidant, antitumor, and anti-inflammatory properties of fungal terpenoids, those derived from *S. crispa* probably exert similar effects. Consequently, this area presents a substantial opportunity for future research to elucidate their specific bioactivities and underlying molecular mechanisms [15,36,37].

### 3.4. Other Bioactive Compounds

In addition to the aforementioned major classes, *S. crispa* contains various other functionally significant molecules. For instance, veratric acid (VA; 3,4-dimethoxybenzoic acid) has been isolated and reported to exert multipotent biological activities, including antioxidant, anti-inflammatory, anti-wrinkle, and photoprotective effects on human skin [38,39]. Researchers also isolated adenosine and specific aromatic compounds (sparaside A, sparalides A–C), which effectively downregulated PCSK9 mRNA expression, indicating their potential as adjunctive therapeutics for hyperlipidemia by modulating cholesterol synthesis genes and lipid metabolism [31]. Furthermore, a specific water-insoluble fraction (SCF4) isolated from the methanolic extract exhibited potent anti-inflammatory activity [9]. Similarly, three novel phenylpropanoids—hanabiratakelide A, B, and C—along with sparoside A, have demonstrated notable antioxidant, anti-inflammatory, and antitumor efficacies [7,15]. Ultimately, continuous, in-depth characterization of these diverse micronutrients and bioactive fractions is imperative to fully exploit the medicinal value of *S. crispa* and substantiate its application in novel pharmaceuticals and functional foods.

## 4. Health Benefits

### 4.1. Immunomodulatory and Antitumor Effects

#### 4.1.1. Immunomodulatory Effects

Crude extracts of *S. crispa* exhibit robust immunostimulatory properties in animal models. The primary active constituent, β-glucan, activates splenocytes upon oral administration and significantly upregulates the secretion of cytokines (IFN-γ, TNF-α, and IL-12p70) in immunosuppressed mice. It further stimulates leukocytes and the broader immune system by promoting the release of complement fragment C5a [5,18,40]. Additionally, phenolic compounds, such as 5-hydroxy-7-methoxyphthalide, effectively attenuate the synthesis of pro-inflammatory cytokines (IL-12p40, IL-6, and TNF-α) in LPS-stimulated bone marrow-derived dendritic cells. This is achieved by suppressing the activation of the NF-κB and MAPK signaling pathways [32]. Furthermore, ergosterol, a provitamin D_2_, promotes immune cell differentiation following its UV-induced conversion [41].

**Figure 1:**
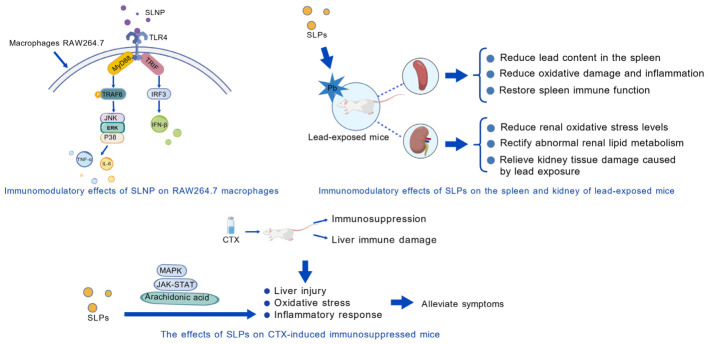
SLNP and SLPs’ immunomodulatory effects and mechanisms. Adapted and summarized from Refs. [20,45,46].

Recent studies have elucidated the complex molecular networks underlying these immunomodulatory effects. Notably, TLR4 has been identified as the primary receptor for *S. crispa* β-glucan via antibody blockade assays, providing a critical target for understanding its mechanism of action [42]. Furthermore, the neutral polysaccharide SLNP activates macrophages via TLR4-mediated dual signaling pathways. Specifically, SLNP upregulates TLR4 expression, initiating the MyD88-dependent pathway. This leads to TRAF6 phosphorylation and subsequent activation of MAPK family members (JNK, ERK, p38), culminating in the release of pro-inflammatory cytokines. Conversely, SLNP also activates IRF3 via a MyD88-independent pathway, promoting type I interferon (IFN-β) production to enhance antiviral responses [20,43]. In the context of physical stress, *S. crispa* counteracts exercise-induced immunosuppression by preventing the downregulation of TLR4/MyD88 protein expression in the small intestine, thereby maintaining mucosal immunity (Figure 1) [44].

Beyond physiological stress, *S. crispa* short-chain polysaccharides (SLPs) offer protective effects against chemically induced immune damage. In cyclophosphamide (CTX)-immunosuppressed mice, SLPs mitigated hepatic injury, oxidative stress, and inflammation by modulating the MAPK and JAK/STAT pathways, alongside arachidonic acid metabolism [45]. Similarly, in lead-exposed models, SLPs effectively reduced splenic lead accumulation, alleviated splenomegaly, and mitigated renal oxidative stress-mediated inflammation and autophagy [46,47]. Moreover, acidic polysaccharides (SCG-A) extracted via hot water have demonstrated direct immunostimulatory activity by promoting rat splenic lymphocyte proliferation in vitro [18]. Based on the findings from previous studies, we constructed the schematic model in Figure 1 to synthesize these mechanisms.

#### 4.1.2. Anti-Inflammatory Effects

**Figure 2:**
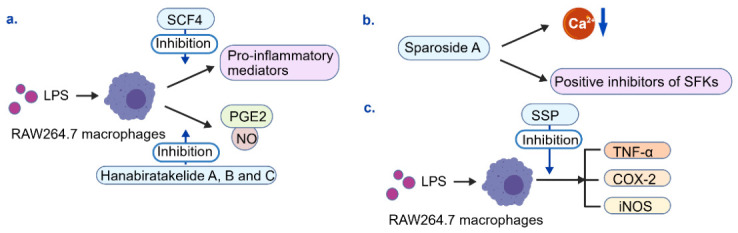
Anti-inflammatory effects of S. crispa. Adapted and summarized from Refs. [9,15,23]. (**a**) Inhibition of pro-inflammatory mediators by SCF4 and hanabiratakelides; (**b**) Reduction of intracellular Ca^2+^ and SFK inhibition by sparoside A. The downward arrow next to Ca^2+^ indicates a reduction; (**c**) Suppression of neuroinflammation by SSP.4.1.3. Antitumor Effects.

The non-aqueous fraction (SCF4) isolated from the methanolic extract of *S. crispa* significantly inhibits pro-inflammatory mediator production in LPS-induced RAW264.7 macrophages. Mechanistically, SCF4 disrupts TLR signaling by inhibiting TAK1 phosphorylation, thereby blocking the downstream activation of the NF-κB and MAPK pathways [9]. Additionally, phthalide derivatives (hanabiratakelide A, B, and C) suppress the production of nitric oxide (NO) and prostaglandin E_2_ (PGE_2_) in LPS-stimulated macrophages [7]. Furthermore, sparoside A dose-dependently reduces intracellular Ca^2+^ concentrations (IC_50_ = 5.06 ± 0.60 μM) and serves as a potent inhibitor of Src family kinases (SFKs), confirming its robust anti-inflammatory capacity [15]. Similarly, *S. subalpina* polysaccharide (SSP) suppresses neuroinflammation-associated gene expression (TNF-α, COX-2, and iNOS) in RAW264.7 cells, highlighting its potential in managing central nervous system disorders (Figure 2) [23].

Various bioactive fractions of *S. crispa* demonstrate substantial antitumor potential [48]. Early studies established that β-(1→3)-D-glucan exerts anti-angiogenic and antimetastatic effects, thereby restricting tumor vascularization and dissemination [49]. Moreover, *S. crispa* selectively inhibits the proliferation of colon cancer cell lines (Caco-2 and Colon-26) and reduces the frequency of azoxymethane-induced aberrant crypt foci (ACF) in vivo [7].

**Figure 3:**
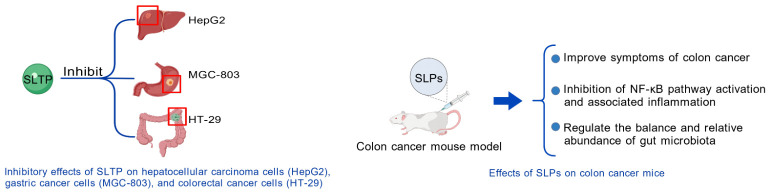
New research progress on the antitumor effects of SLTP and SLPs. Compiled and redrawn based on data from [25,50]. The red boxes highlight the tumor sites.

Recent advancements have further broadened this scope (Figure 3). Total protein extract (SLTP) significantly suppresses the in vitro proliferation of gastrointestinal cancer cell lines, including HepG2, MGC-803, and HT-29 [25]. In vivo, SLPs demonstrated efficacy in an AOM/DSS-induced colon cancer model by reducing disease activity indices, tumor multiplicity, and tumor size. This efficacy is attributed to a synergistic modulation of immune responses, signaling pathways, and the gut microbiota [50]. In vitro studies have shown that crude polysaccharides from *S. crispa* inhibit the proliferation of human colon cancer cell lines, with long-term consumption potentially reducing the risk of colon cancer [51]. Furthermore, ethanolic extracts rich in triterpenoids (SLEE, 11.40% content) exhibited concentration-dependent cytotoxicity against MGC-803 and HT-29 cells [25]. In the realm of biotherapeutics, *S. crispa* β-glucan has been shown to synergize with antibody drugs against lymphoma cells by upregulating natural killer (NK) cell activity, thereby enhancing antibody-dependent cell-mediated cytotoxicity (ADCC) [52]. Lastly, the chloroform fraction (CESP) selectively targets cervical cancer stem cells (HeLa-derived), inducing apoptosis and suppressing tumor sphere formation, with robust tumor inhibition verified in a chick embryo chorioallantoic membrane (CAM) model [53].

### 4.2. Anti-Oxidant and Neuroprotection Effects

#### 4.2.1. Anti-Oxidant Effects

**Figure 4:**
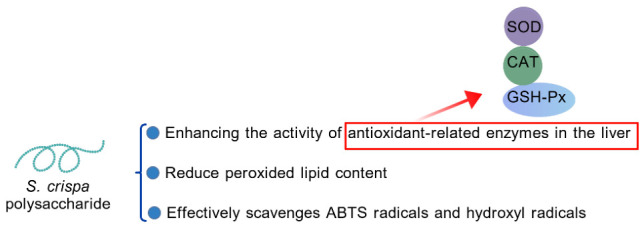
Recent advances in the primary antioxidant effects of *S. crispa* polysaccharides. Compiled and redrawn based on data from [56,58].

*S. crispa* possesses a robust antioxidant profile critical for mitigating oxidative stress. Comparative studies indicate that *S. crispa* exhibits higher DPPH radical scavenging activity and total phenolic content than *Lentinus edodes* and *Mycoleptodonoides aitchisonii* [54]. Studies have confirmed that phenolic compounds in *S. crispa* (Hanabiratakelide A, B, and C) show superoxide dismutase-like activity stronger than vitamin C [7]. Polysaccharides, the primary natural antioxidants in *S. crispa*, display strong free radical scavenging capabilities. Research indicates an inverse relationship between polysaccharide molecular weight and antioxidant capacity [55]. For instance, a specific polysaccharide fraction (SCP) achieved a 94.83% DPPH scavenging rate at 8 mg/mL, comparable to vitamin C, while effectively neutralizing hydroxyl (·OH) and superoxide (O_2_^−^·) radicals [56,57]. In vivo, SLPs ameliorated lipid peroxidation in rats fed a high-fat/high-cholesterol diet by significantly upregulating hepatic antioxidant enzymes (SOD, CAT, and GSH-Px) and reducing malondialdehyde (MDA) levels [58]. Beyond polysaccharides, *S. crispa* glycoproteins demonstrate dose-dependent radical scavenging alongside excellent functional properties (e.g., emulsifying and foaming stability), making them promising multifunctional food additives [26]. Figure 4 outlines these antioxidant mechanisms.

#### 4.2.2. Neuroprotective Effects

**Figure 5:**
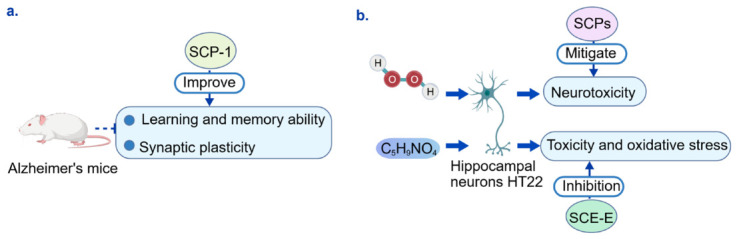
Neuroprotective effects of S. crispa. Compiled and redrawn based on data from [59,61]. (**a**) Mitigation of cognitive deficits and improvement of synaptic plasticity by SCP-1; (**b**) Mitigation of H_2_O_2_-induced neurotoxicity by SCPs, and inhibition of glutamate-induced toxicity and oxidative stress by SCE-E.

Emerging evidence highlights the neuroprotective potential of *S. crispa* (Figure 5). Polysaccharide SCP-1 mitigates cognitive deficits and improves synaptic plasticity in Alzheimer’s disease mouse models, concurrently modulating the gut microbiota and suppressing neuroinflammation [59]. In vitro, ultrasound-assisted enzyme-extracted polysaccharides (SCPs) protect hippocampal HT22 cells against H_2_O_2_-induced neurotoxicity by enhancing cell viability, reducing lactate dehydrogenase (LDH) leakage, and lowering intracellular ROS levels [60]. Similarly, the ethanolic extract (SCE-E) protects HT22 cells from glutamate-induced excitotoxicity and oxidative stress. Mechanistically, SCE-E activates the Nrf2 and CREB pathways via AKT and ERK phosphorylation, presenting a promising dietary intervention for neurodegenerative conditions [61].

### 4.3. Metabolic Regulation

#### 4.3.1. Anti-Hypertensive and Hypolipidemic Effects

*S. latifolia* polysaccharides (SLPs) regulate cholesterol metabolism by repairing intestinal barriers, modulating cholesterol-related proteins, and enriching short-chain fatty acid (SCFA)-producing bacteria in hyperlipidemic rats [62]. For hypertension, long-term consumption of *S. crispa* improves endothelial function and survival in stroke-prone spontaneously hypertensive rats. This is mediated by the restoration of Akt-dependent phosphorylation of endothelial nitric oxide synthase (eNOS), subsequently increasing NO bioavailability [13]. Furthermore, specific ergosterol derivatives act as potent inhibitors of PCSK9 mRNA expression, indicating utility as adjunctive therapeutics alongside statins for hyperlipidemia [31].

#### 4.3.2. Hypoglycemic Effect

Dietary intervention with *S. crispa* effectively ameliorates type 2 diabetes and associated cardiometabolic disorders in obese murine models. The supplementation significantly elevates plasma adiponectin levels, which in turn reduces adipocyte hypertrophy and improves systemic glucose metabolism, ultimately decreasing fasting blood glucose, insulin, triglycerides, and cholesterol levels [63].

#### 4.3.3. Anti-Obesity Effects

**Figure 6:**
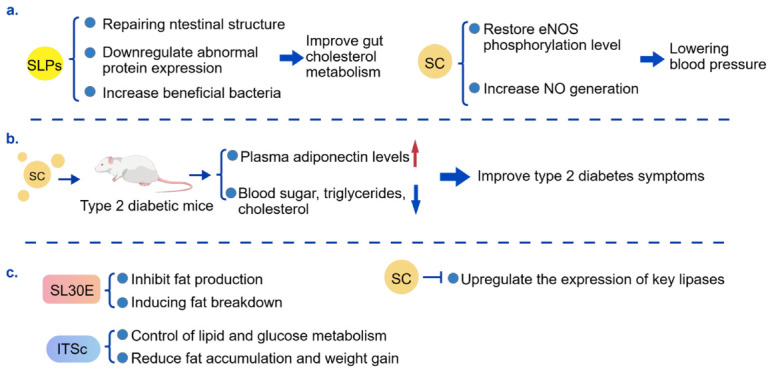
Metabolic regulation effects of *S. crispa.* Adapted and summarized from Refs. [62,63,64]. (**a**) Anti-hypertensive and hypolipidemic effects: SLPs regulate cholesterol, SC improves endothelial function, and ergosterol derivatives inhibit PCSK9. (**b**) Hypoglycemic effect: SC ameliorates diabetes by elevating adiponectin and improving glucose metabolism. Red upward arrows indicate an increase, and blue downward arrows indicate a decrease. (**c**) Anti-obesity effects: SL30E reduces lipid accumulation, SC mitigates weight gain, and ITSc prevents fat accumulation.

*S. crispa* exerts significant anti-obesity effects through multiple pathways. In vitro, a 30% ethanolic extract (SL30E) attenuated lipid accumulation in 3T3-L1 adipocytes by downregulating adipogenesis while promoting lipolysis, lipophagy, and thermogenesis [64]. In vivo, aqueous extracts mitigated weight gain in high-fat diet-induced obese mice by upregulating key lipolytic enzymes [65]. Additionally, the powdered fruiting bodies of indoor-cultivated *S. crispa* (ITSc) prevented anomalous fat accumulation and weight gain in ovariectomized mice, suggesting its potential to alleviate menopause-associated metabolic dysregulation [66], contributing to the overall metabolic regulatory network summarized in Figure 6.

### 4.4. Microbiota Modulation

**Figure 7:**
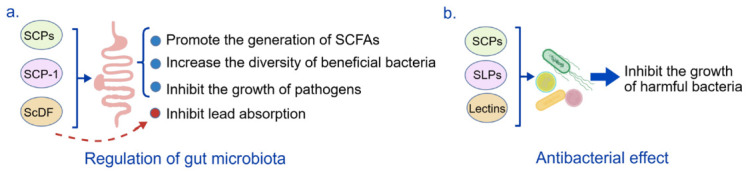
Microbiota modulation effects of S. crispa. Compiled and redrawn based on data from [67,69]. (**a**) Regulation of gut microbiota: promotion of beneficial taxa and SCFA production, alongside pathogen inhibition by SCPs, SCP-1, and ScDF. The blue arrows indicate common regulatory effects. The red dashed arrow represents a unique effect specific to ScDF, which limits intestinal lead absorption. (**b**) Antibacterial effect: direct antimicrobial activities against various pathogenic microbes by *S. crispa* compounds (SCPs, SLPs, and lectins).

The prebiotic properties of *S. crispa* are pivotal to its health benefits. Figure 7 depicts these microbiota-modulating effects. In vitro fermentation models reveal that gut microbiota selectively utilize *S. crispa* polysaccharides (SCPs and SCP-1) to produce SCFAs, thereby promoting the proliferation of beneficial taxa while inhibiting opportunistic pathogens. Predictive functional profiling suggests that these polysaccharides significantly enhance microbial carbohydrate, amino acid, and energy metabolism [67,68]. Furthermore, *S. crispa* soluble dietary fiber (ScDF) mitigates lead toxicity by modulating the gut microbiome. ScDF limits intestinal lead absorption, promotes its excretion, and fosters a microbial environment that increases SCFA production while inhibiting Helicobacter pylori colonization [24].

Beyond prebiotic effects, *S. crispa* compounds exhibit direct antimicrobial activity. SCPs disrupt glycolysis and the tricarboxylic acid (TCA) cycle in Staphylococcus aureus, inducing fatal metabolic dysfunction [22]. In addition to polysaccharides, lectins from *S. crispa* also demonstrate antibacterial and antifungal potential. Activity assays show that purified lectins effectively inhibit drug-resistant bacteria (e.g., *Escherichia coli*, *Staphylococcus aureus*, *Pseudomonas aeruginosa*) and fungi (e.g., *Candida* and *Fusarium* species). These findings suggest that *S. crispa* lectins have potential as novel therapeutic agents [25]. In oral health applications, SLPs combined with artificial saliva significantly suppress the proliferation of cariogenic bacteria (*Streptococcus mutans*, *S. salivarius*, and *S. sanguinis*) [69].

### 4.5. Other Health-Related Effects

**Figure 8:**
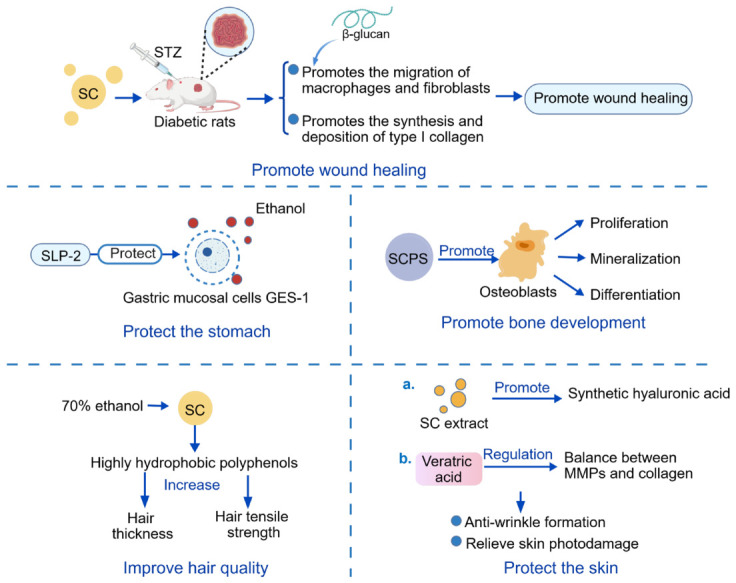
Other health benefits of *S. crispa.* Compiled and redrawn based on data from [19,70,72]. (**a**) Moisturizing properties of *S. crispa*. (**b**) Anti-photoaging effects of its active components.

As summarized in Figure 8, the therapeutic repertoire of *S. crispa* extends to several other physiological domains.

In tissue regeneration, oral administration of β-glucans accelerates diabetic wound healing. It promotes the migration of macrophages and fibroblasts and upregulates type I collagen synthesis, resulting in a 40% increase in collagen deposition at the wound site in STZ-induced diabetic rats [11,70]. In gastrointestinal care, prophylactic treatment with polysaccharide SLP-2 markedly protects gastric mucosal cells (GES-1) against ethanol-induced injury [19]. For skeletal health, water-soluble polysaccharides (SCPS) stimulate the proliferation and mineralization of pre-osteoblast MC3T3-E1 cells, indicating osteogenic potential [21].

In dermatological applications, *S. crispa* extracts exhibit potent moisturizing properties by upregulating hyaluronic acid synthase genes (HAS1, HAS2, and HAS3) [71]. Concurrently, veratric acid mitigates UV-induced photoaging and wrinkle formation by suppressing matrix metalloproteinase (MMP) expression and boosting type I collagen and TIMP production, thereby preserving the extracellular matrix [72]. Finally, highly hydrophobic polyphenols derived from ethanolic extracts significantly enhance hair diameter, tensile strength, and cuticular morphology [33].

Because the history of artificial cultivation for the rare edible mushroom *S. crispa* is less than 30 years, human clinical trials investigating its pharmacological effects are currently relatively scarce. Therefore, the aforementioned findings regarding its pharmacological activities, which are based on in vitro and animal studies, still require further validation through clinical trials. Table 2 shows the active ingredients and efficacy of *S. crispa*. Data primarily from *S. crispa* and closely related taxa (*S. latifolia*, *S. subalpina*).

## 5. Cultivation and Commercialization

### 5.1. Biological Characteristics

From a cultivation perspective, *S. crispa* sensu lato is highly sensitive to environmental factors. The suitable temperature range for mycelial growth is 20–25 °C, with an optimum at 25 °C; growth markedly decreases above 30 °C. The optimal pH is generally between 5.0 and 7.0, with the best growth observed at pH 6 [73]. The moisture content of the substrate is usually controlled around 60–65%. As an aerobic fungus, it requires moderate ventilation during the mycelial growth stage, while fruiting body development requires sufficient light exposure, and red or natural scattered light is considered beneficial for morphological development [1,73]. Compared with the most common edible mushrooms, *S. crispa* sensu lato exhibits slower mycelial growth rates and a stronger dependence on nutrient supply and ecological conditions, resulting in a relatively long growth cycle. These characteristics increase the difficulty of factory cultivation to a certain extent and impose stricter requirements on substrate type, moisture content, and environmental stability. Existing studies indicate that its biological characteristics are significantly influenced by the interaction of strain genetics, substrate composition, and environmental factors, with notable phenotypic variations among strains from different sources. More systematic comparative studies are still needed to optimize cultivation parameters [73,74].

### 5.2. Cultivation Development History

Japan was one of the first countries to successfully develop artificial cultivation technology and achieve commercialization, and South Korea also conducted early research and realized commercial production [4]. China quickly followed suit, making significant breakthroughs in factory-scale cultivation. According to data from the China Edible Fungi Association (CEFA), China’s *Sparassis* production reached 12,826 tons in 2024, with Fujian and Hainan provinces emerging as the major domestic production areas [74,75]. Molecular identification studies have shown that most Asian cultivated strains belong to *S. latifolia*, which differs morphologically and genetically from the European/North American *S. crispa*, although their bioactive profiles are highly similar, allowing for valuable data cross-referencing [3,76,77]. In recent years, remarkable progress has been made in the breeding of elite strains and the optimization of cultivation substrates. For instance, factory-specific cultivated strains of *S. latifolia*, such as Xiu C (SP-C) and Minxiu No. 1, exhibit excellent agronomic traits, including uniform fruiting, optimal petal expansion, robust contamination resistance, and high biological efficiency [78,79]. Benefiting from these technological breakthroughs, the standardization and yield stability of China’s factory-scale *Sparassis* production have reached an internationally advanced level.

### 5.3. Factory Cultivation

Factory cultivation primarily adopts bag or bottle systems, with the core challenges lying in substrate optimization, precise environmental control, and contamination prevention. The commonly used substrate is based on coniferous sawdust (particularly larch sawdust) (55–60%), supplemented with cottonseed hulls (20–25%), wheat bran (15–18%), corn flour (2–3%), gypsum (1%), and lime (0.5%). Specific ratios should be adjusted according to raw material quality and strain characteristics [1,80]. The cultivation process generally includes substrate preparation and mixing, bagging, autoclaving, cooling and inoculation, spawn running, primordia induction and fruiting management, and harvesting. During the mycelial running phase (40–60 days post-inoculation), the temperature is typically maintained at 23–25 °C under dark or low-light conditions [73]. During the fruiting stage (60–115 days post-inoculation)—which consists of primordia induction (25–30 days) and fruiting body development (25–30 days)—the temperature is lowered while relative humidity is kept high. Additionally, sufficient illumination and enhanced ventilation are provided to prevent CO_2_ accumulation and subsequent fruiting body malformation. The entire cultivation cycle from inoculation to harvest is relatively long (95–115 days), with biological efficiency varying depending on the strain, substrate composition, and the level of environmental control [1,80]. Although mechanized mixing and environmental control systems have improved efficiency to some extent, the environmental sensitivity of *S. crispa* sensu lato, elevated contamination risk, and long growth cycle result in relatively high production costs [1]. The effects of key parameters such as substrate formulation, moisture content, and light management on the accumulation of bioactive compounds (e.g., β-glucans) have not yet been fully elucidated and require more targeted research [6,74]. In summary, despite the successful advancements of industrial cultivation in terms of standardization, yield consistency, and economic feasibility, considerable room for optimization remains. Future endeavors should prioritize the development of intelligent, precision-control technologies and foster integrated research bridging cultivation and downstream processing, thereby driving the sustainable development of the industry [1,74].

## 6. Processing Technologies

**Figure 9:**
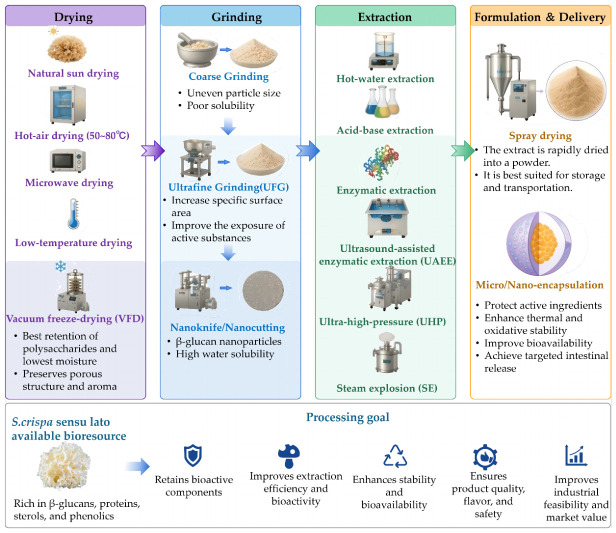
Effects of different processing unit operations on the quality and bioactive retention of *S. crispa*.

*S. crispa* sensu lato represents a highly valuable bioresource, rich in β-glucans, proteins, sterols, and phenolics, offering substantial commercial potential in functional foods, nutraceuticals, and cosmetics. Post-harvest processing methods directly determine the retention of nutritional components, functional properties, and the ultimate market value of the products. The main processing unit operations include drying, grinding, extraction, and formulation technologies (Figure 9). Different techniques show significant variations in the retention of heat-sensitive components (such as polysaccharides and phenolics), energy consumption, extraction efficiency, and overall product quality.

### 6.1. Drying Technologies

Drying is a critical preservation step that significantly impacts the bioactive profile of *S. crispa*. Common drying methods include natural sun drying, hot-air drying, microwave drying, low-temperature drying, and vacuum freeze-drying (VFD). Hot-air drying (typically 50–80 °C) is widely adopted due to its simplicity and low cost. However, prolonged exposure to elevated temperatures often leads to a decrease in polysaccharide molecular weight and bioactivity, protein denaturation, vitamin C oxidation, and structural degradation of phenolic compounds [81].

Comparative studies showed that vacuum freeze-drying (VFD) exhibited the best performance in polysaccharide retention, with a content of 34.8% and the lowest residual moisture (2.66%), significantly outperforming microwave and high-temperature drying [81]. VFD preserves the porous microstructure of the cellular matrix through ice crystal sublimation under vacuum, thereby avoiding thermal degradation. Pre-freezing fresh fruiting bodies at −80 °C for 24 h followed by 48 h VFD effectively retains volatile aromatic compounds, resulting in a flavor profile close to that of fresh *S. crispa* [82].

### 6.2. Grinding Technology

Grinding improves the convenience and applicability of *S. crispa* in food formulations. Conventional coarse pulverization is simple and requires no specialized equipment, but it often results in uneven particle size distribution, limited solubility, and localized heat generation that accelerates the oxidation of sensitive bioactives [83,84].

Ultrafine grinding (UFG) significantly enhances the physicochemical properties of *S. crispa* powder by increasing the specific surface area and exposing internal bioactive sites [85,86]. For example, using a planetary ball mill at 300 r/min for 5 min effectively reduces particle size, increases specific surface area, and exposes more bioactive sites, thereby improving total phenolic content, DPPH, ABTS^+^, and hydroxyl radical scavenging rates, as well as water-holding capacity and solubility [87]. UFG-assisted processing can achieve a polysaccharide yield of 20.80% and favors the release of high-molecular-weight fractions, although further optimization is still needed for selective β-glucan extraction [88]. Innovative mechanical shearing techniques, such as the insoluble tungsten carbide nanoknife, can achieve a β-glucan extraction rate of 70.2%, producing β-(1→3)-D-glucan nanoparticles (~150 nm) with excellent water solubility (approximately 90% after standing for 10 min) [89].

### 6.3. Extraction Technologies

The extraction process profoundly affects the molecular weight, degree of branching, functional groups, and biological activities of polysaccharides [90]. Hot-water extraction (50–100 °C, 1.5–5 h) is simple and low-cost, but it is time-consuming, energy-intensive, and prone to causing thermal degradation of active components [91,92,93]. Acid-base extraction (using NaOH, KOH, or HCl) disrupts hydrogen bonds and ester linkages in the cell wall [94]. For instance, KOH extraction can achieve a β-glucan purity of up to 87.96%, although extreme pH and high temperature may alter the native structure of polysaccharides [88,95].

Enzymatic extraction using cellulase (optimized conditions: 1.3% enzyme dosage, 40 °C, 2.5 h, pH 5.5) yields 22.90 ± 0.26% polysaccharides, significantly higher than hot-water extraction [96]. Ultrasound-assisted enzymatic extraction (UAEE) enhances enzyme penetration through acoustic cavitation, increasing the yield of *S. crispa* polysaccharides (SCPs) to 14.63% (a 68.54% improvement over conventional methods) while favorably modulating the molecular weight distribution [60]. Ultra-high-pressure (UHP) extraction (optimized conditions: 315 MPa, pH 9.2, 48.5 °C, 15 min) achieves a polysaccharide yield of 14.72 ± 0.15% by disrupting non-covalent interactions in cell membranes under mild conditions [97]. Steam explosion (SE) pretreatment (225 °C, 5 min) followed by instantaneous decompression physically disrupts the lignocellulosic matrix, increasing the β-(1→3)-glucan yield by approximately 1.6-fold, making it suitable for large-scale industrialization [91]. For high-molecular-weight polysaccharides with limited solubility, controlled depolymerization methods (ultrasound, hydrochloric acid, or H_2_O_2_/Vc) can produce lower-molecular-weight fractions without changing monosaccharide composition. The H_2_O_2_/Vc method, in particular, generates hydroxyl radicals and yields fractions with enhanced antioxidant activity, as validated in zebrafish models [98,99].

### 6.4. Formulation and Delivery Technologies

To improve the stability, solubility, bioavailability, and targeted delivery of bioactive compounds from *S. crispa* (particularly β-glucans and phenolics), various formulation and delivery technologies have been explored. Among these, spray-drying and micro/nano-encapsulation are the most widely applied due to their scalability and cost-effectiveness.

However, conventional spray-drying of *S. crispa* aqueous extracts often faces processing challenges, such as the high viscosity of its high-molecular-weight β-glucans and the risk of thermal degradation at high inlet temperatures [81,94]. To overcome these limitations, advanced encapsulation technologies are increasingly utilized to provide a protective physical barrier against harsh gastrointestinal environments. For example, *S. crispa* β-glucan microcapsules prepared with a maltodextrin: whey protein isolate (1:2) wall material showed a threefold higher thermal decomposition temperature and excellent targeted intestinal release (only 12% released in simulated gastric fluid vs. 85% in intestinal fluid) [100]. This targeted delivery is crucial, as the gut is the primary site where *S. crispa* polysaccharides exert their prebiotic and immunomodulatory effects [67,68].

Despite these promising data, the current literature on *S. crispa* processing heavily emphasizes upstream extraction, leaving downstream delivery systems underexplored. Future industrial processing must critically evaluate the cost-effectiveness of complex encapsulation matrices. A promising future trajectory involves integrating novel physical treatments directly with advanced delivery systems to maximize the commercial viability of *S. crispa* functional foods.

## 7. Challenges and Strategies for Industrial Development

### 7.1. Current Development Status

Due to its unique morphology, comprehensive nutritional profile, and profound pharmacological effects, *S. crispa* has garnered increasing popularity across global markets, particularly in South Korea, China, Japan, Germany, and the United States. As a dual-purpose fungus (edible and medicinal), its industrial trajectory is primarily driven by the extraction of active ingredients and continuous innovation in product formulation. Currently, primary processed products—such as fresh, dried, and pickled *S. crispa*—are widely circulated. However, the commercial landscape for deep-processed products is still evolving, with primary applications concentrated in functional foods, dietary supplements, nutraceuticals, and cosmeceuticals. Commercially, deep-processed *S. crispa* is predominantly formulated into convenient delivery systems. Recent market trends indicate a growing diversity in *S. crispa* deep-processed products, such as functional beverages, gummy supplements, and microencapsulated powders, reflecting the increasing consumer demand for its health-promoting properties.

### 7.2. Challenges

*S. crispa* is highly valued for its nutritional and functional properties, but its processing faces the challenge of “high raw material value, low product added value” due to limited deep processing. This issue arises from unstable raw material supply, inefficient active ingredient extraction, insufficient research on processing technologies, and a lack of high-added-value products. Current research mainly focuses on extracting nutritional components, while drying methods, crucial for processing quality, functionality, and shelf life, remain underexplored. Existing studies primarily discuss conventional drying techniques, with limited research on the degradation of functional components like polysaccharides and phenolic compounds, or the relationship between drying parameters and quality indicators such as texture and rehydration. Drying methods for other edible fungi, such as heat pump, microwave, and infrared drying, offer insights for future *S. crispa* drying technologies.

Firstly, the supply of raw materials for *S. crispa* is unstable. The growth cycle of *S. crispa* is relatively long [101]. Moreover, during artificial cultivation, strict control of temperature, humidity, ventilation, and ensuring sufficient light is required, which imposes high demands on the cultivation environment [73]. This results in low yields and inconsistent quality in the artificial cultivation of *S. crispa*. Furthermore, the development of active ingredients from *S. crispa* mainly remains at the raw material extraction stage, with insufficient technology for converting these into high-value-added functional products. For example, although β-glucan has been confirmed to have immune-regulating, anti-tumor, and other beneficial effects, its formulation into oral preparations faces challenges such as low bioavailability and poor stability within the human body [102]. The extraction efficiency of active ingredients from *S. crispa* is relatively low. The fruiting bodies of *S. crispa* are primarily composed of fungal cell walls, which have a dense structure [103].

Additionally, although β-glucan in *S. crispa* is abundant and predominantly bound, traditional extraction methods fail to disrupt cell walls effectively, while advanced technologies remain immature for industrial use. Antibacterial metabolites inhibit enzymatic hydrolysis efficiency, and non-standardized ultrasonic extraction causes yield fluctuations, limiting stable high-purity polysaccharide supply. Moreover, abundant SOD readily loses activity during processing owing to inadequate preservation and drying techniques. Deep-processing research is fragmented, focusing mainly on cultivation with limited studies on extraction, fermentation, and formulation; gaps in β-glucan degradation and flavor-compound dynamics during drying and fermentation provide insufficient theoretical support for optimization. Modern approaches such as online monitoring and intelligent fermentation are virtually absent, hindering precise control and industrial upgrading. Currently, the deep processing products of *S. crispa* on the market are relatively limited, and there is insufficient innovation in deep processing technology research. *S. crispa* is rich in β-glucan, possesses various pharmacological activities that promote health, and its nutritional value can be enhanced through fermentation. The polysaccharides from *S. crispa* have unique rheological and gelling properties, making them potentially useful in the food industry. Furthermore, *S. crispa* also exhibits high DPPH scavenging activity and acetylcholinesterase inhibition activity. There is a need to fully utilize the pharmacological activities and processing characteristics of *S. crispa* to innovate deep processing technologies and develop diversified products. In addition, the industrial development of *S. crispa* faces challenges such as incomplete preservation and transportation technologies, a lack of standardized regulations, and low market awareness.

### 7.3. Strategies

To promote the industrialization of *S. crispa*, it is necessary to strengthen innovation in processing technologies and upgrade production models. In terms of raw material supply, the promotion of intelligent cultivation technology should be encouraged. For example, the Zhejiang Academy of Agricultural Sciences in China has partnered with well-known enterprises to jointly develop a movable “5G Smart Mushroom House” tailored to the growth characteristics of *S. crispa*. This integrates artificial intelligence, the Internet of Things (IoT), and biotechnology to control the temperature and humidity within suitable ranges and simulate lighting with LED strips, achieving energy-saving, emission reduction, and increased yield.

To address the issues of insufficient active substance extraction and significant batch-to-batch product variability, the industry must transition from crude processing to precision processing. Research should deeply explore the synergistic mechanisms among various active ingredients to develop evidence-based compound functional products. Crucially, a rigorous and standardized quality control system must be established. Specifically, Critical Control Points (CCPs) must be implemented during processing, such as strictly monitoring drying temperatures and extraction pH, to prevent the thermal and chemical degradation of heat-sensitive polysaccharides and phenolics. Furthermore, potential contaminants must be rigorously screened, including heavy metal accumulation from cultivation substrates, mycotoxins, and excessive microbial loads during prolonged storage. For the validation of final products, key parameters such as residual moisture content, specific β-glucan concentration, and molecular weight distribution must be standardized as core quality indicators to ensure consistent biological efficacy. Building upon these quality assurances, the development of high-value-added products—ranging from ready-to-eat functional soups to dietary supplements and cosmetics—will effectively enhance market competitiveness. Finally, converting processing residues into animal feed, fertilizers, or bioenergy will establish a “zero-waste”, positive-cycle bioeconomy for the *S. crispa* industry.

## 8. Conclusions and Future Perspective

*S. crispa* is a high-value edible and medicinal fungus rich in β-glucans, proteins, sterols, phenolics and other components. These constituents demonstrate widespread biological activities, including immune modulation, antitumor, antioxidant, neuroprotection, and metabolic regulation. Especially in immune regulation and gut microbiota–immune axis modulation, clear target pathways have been identified, such as activation through the TLR4–MyD88/TRIF dual pathway. In terms of technological innovation, methods such as vacuum freeze-drying, enzyme–ultrasound synergistic extraction, and microencapsulation have significantly improved the yield, stability, and functional retention of active components, providing a technological foundation for the development of high-value products.

In the future, we can select high-quality strains through breeding and cultivation techniques and promote intelligent environmental control systems to ensure a stable supply of *S. crispa* raw materials in terms of both quantity and quality. We should further explore the mechanisms of active components and strengthen the research on the synergistic effects between *S. crispa* polysaccharides, terpenes, polyphenols, and other components, expanding their application prospects. At the same time, we need to strengthen the innovation and integration of processing technologies, promote the development of green and efficient novel extraction methods, facilitate the development of high-value products, extend the deep-processing industrial chain of *S. crispa*, and promote the sustainable development of the *S. crispa* industry. Future interdisciplinary collaboration and industrial chain integration are expected to broaden its applications and enhance its value in the food, pharmaceutical, and health sectors.

## Figures and Tables

**Table 1:** Recent discoveries of polysaccharides from *Sparassis* species. *S. crispa*, *Sparassis crispa*; *S. latifolia*, *Sparassis latifolia*; *S. subalpina*, *Sparassis subalpina*.

Polysaccharide	Source	Monosaccharide Composition (Molar Ratio)	Structural Features	Ref.
SCP-1	*S. crispa*	Glc:Gal:Fuc:Man (52.10:31.10:15.04:1.76)	Backbone: (1→6)-α-D-Galp, (1→6)-β-D-Glcp, (1→3)-β-D-Glcp	[2]
SCG-A	*S. crispa*	Mainly Glc and Gal, minor Man and GalA	Backbone: α-(1→4)-D-glucan with (1→6) branches	[18]
SLP-2	*S. latifolia*	____	Backbone: →4)-α-D-Glcp-(1→, →3)-α-D-Glcp-(1→, →3,4)-α-D-Glcp-(1→	[19]
SLNP	*S. latifolia*	Ara:Gal:Glc:Xyl:Man (6:12:63:10:5)	Neutral pyranose-type polysaccharide	[20]
SCPS	*S. crispa*	Ten different monosaccharides and uronic acids	Water-soluble polysaccharide	[21]
SCPs	*S. crispa*	Fuc:Glc:Gal (0.043:0.652:0.305)	____	[22]
SSP	*S. subalpina*	Glc:Man:Gal:Ara (6.5:1.3:1:1)	Backbone: →3)-α-D-Galp-(1→2)-β-D-Glcp-(1→3)-β-L-Araf-(1→3)-α-D-Manp-(1→	[23]
ScDF	*S. crispa*	Uronic acid-rich	Backbone: β-(1→3,6)-glucan with glucuronic acid side chains	[24]

**Table 2:** Recent advances in the active components and efficacy of *S. crispa*.

Category	Active Ingredient	Health Benefits	Refs.
Polysaccharide	*S. crispa* polysaccharide (SCP-1)	Regulates gut microbiota, has anti-microbial effects, suppresses inflammation, provides neuroprotection, and prevents Alzheimer’s disease.	[59,68]
	Water-soluble *S. crispa* polysaccharide (SCPS)	Promotes osteoblast proliferation and differentiation, potentially preventing osteoporosis.	[21]
	*S. latifolia* neutral polysaccharide (SLNP)	Immune-modulating effects, anti-cancer.	[20]
	*S. latifolia* polysaccharide (SLP)	Immune-modulating effects, anti-inflammatory, anti-oxidant, and regulates cholesterol metabolism.	[58,62]
	*S. latifolia* polysaccharides (SLP-2)	Gastric protective effect.	[19]
	*S. latifolia* polysaccharides (SLPs)	Regulates gut microbiota, has anti-inflammatory and anti-cancer effects, immune modulation, promotes lead excretion, and has anti-bacterial properties.	[45,50,69]
	β-Glucans	Immune-modulating effects, anti-cancer effects, and promotion of wound healing.	[70]
	*S. crispa* polysaccharides (SCPs)	Regulate gut microbiota, anti-bacterial, and neuroprotective effects.	[22,60]
	*S. subalpina* polysaccharide(SSP)	Anti-inflammatory	[23]
	*S. crispa* soluble dietary fiber (ScDF)	Antagonize lead toxicity, regulate gut microbiota, and repair intestinal damage.	[24]
Protein	Total protein of *S. latifolia* (SLTP)	Anti-cancer effect.	[25]
	*S. crispa* glycoprotein	Antioxidant effect.	[26]
	Lectin	Antimicrobial effect.	[27]
Ergosterol	Peroxymicronostatin	Anti-inflammatory, anti-oxidant, anti-tumor, and anti-complement effects.	[30]
	Ergosterol derivatives	Improve hyperlipidemia.	[31]
Phenolic	phenolic compounds	Anti-oxidant effect.	[10]
	5-hydroxy-7-methoxyphthalide	Immune-modulating effects, anti-inflammatory.	[32]
	high hydrophobic polyphenol	Improve hair quality.	[33]
Terpenes	Sesquiterpenoid	Anti-oxidant and anti-bacterial activities.	[35]
	Triterpenoid compounds	Potential anti-cancer properties.	[25]
Other	Veratric acid	Anti-wrinkle, preventing and treating photoaging of the skin.	[72]
	Phthalic acid derivatives (hanabiratakelide A, B, and C)	Anti-oxidant, anti-inflammatory, and anti-tumor effects.	[7]
	sparoside A	Anti-inflammatory.	[15]
	Sparaside A, Sparalides A-C, Adenosine	Lower cholesterol, regulating lipid metabolism.	[31]
	The extract of *S. crispa*	Moisturizing effect.	[71]
	*S. latifolia*’s 30% ethanol extract (SL30E)	Anti-obesity effect.	[64]
	*S. crispa* ethanol extract (SCE-E)	Neuroprotective effect.	[61]
	The fruiting body powder of the indoor cultivation of *S. crispa* (ITSC)	Anti-obesity effects, alleviating menopausal symptoms.	[66]
	*S. crispa* non-aqueous substances in methanol extracts(SCF4)	Anti-inflammatory effect.	[9]

## Data Availability

No new data were created or analyzed in this study. Data sharing is not applicable to this article.
